# The Ability and Mechanism of nHAC/CGF in Promoting Osteogenesis and Repairing Mandibular Defects

**DOI:** 10.3390/nano12020212

**Published:** 2022-01-10

**Authors:** Yuhe Zhu, Nanjue Cao, Yue Zhang, Guangxiu Cao, Chunping Hao, Keda Liu, Xiaoming Li, Wei Wang

**Affiliations:** 1 Liaoning Provincial Key Laboratory of Oral Diseases, School and Hospital of Stomatology, China Medical University, Shenyang 110001, China; yhzhu74@cmu.edu.cn (Y.Z.); njcao@zju.edu.cn (N.C.); yzhang93@cmu.edu.cn (Y.Z.); 2019121274@cmu.edu.cn (C.H.); kdliu@cmu.edu.cn (K.L.); 2 Key Laboratory for Biomechanics and Mechanobiology of Ministry of Education, Beijing Advanced Innovation Center for Biomedical Engineering, School of Biological Science and Medical Engineering, Beihang University, Beijing 100083, China; by1810102@buaa.edu.cn

**Keywords:** concentrated growth factor, mineralized collagen, bone defect, bone regeneration

## Abstract

Nano-hydroxyapatite/collagen (nHAC) is a new type of bone tissue engineering scaffold material. To speed up the new bone formation of nHAC, this study used concentrated growth factor (CGF) and nHAC in combination to repair rabbit mandibular defects. nHAC/CGF and nHAC were implanted into rabbit mandibles, and X-ray, Micro-CT, HE and Masson staining, immunohistochemical staining and biomechanical testing were performed at 8, 16 and 24 weeks after surgery. The results showed that as the material degraded, the rate of new bone formation in the nHAC/CGF group was better than that in the nHAC group. The results of the HE and Masson staining showed that the bone continuity or maturity of the nHAC/CGF group was better than that of the nHAC group. Immunohistochemical staining showed that OCN expression gradually increased with time. The nHAC/CGF group showed significantly higher BMP2 than the nHAC group at 8 weeks and the difference gradually decreased with time. The biomechanical test showed that the compressive strength and elastic modulus of the nHAC/CGF group were higher than those of the nHAC group. The results suggest that nHAC/CGF materials can promote new bone formation, providing new ideas for the application of bone tissue engineering scaffold materials in oral clinics.

## 1. Introduction

In the field of oral implantation, tooth loss is often accompanied by trauma, inflammation, cysts, and periodontitis causing horizontal or vertical defects of the alveolar ridge. Bone deficiency is an urgent problem in the field of implants [1]. Currently, the most common solution is to increase bone mass by implanting bone tissue engineering scaffold materials into the bone defect. Commonly used bone graft materials include autogenous bone and xenogeneic bone [2]. The disadvantages of autogenous bone, such as limit source and complex surgery procedure, hinder its application [3]. Synthetic biomaterials are widely researched and applied to solve this problem [4,5].

Nano-hydroxyapatite/collagen (nHAC) is an artificial bone repair material developed in recent years [6]. It is a three-dimensional porous bone tissue engineering scaffold material composed of nano-hydroxyapatite (nano-HA) and type I collagen (COL), which features a chemical composition and microstructure similar to the human body’s natural bone matrix [7]. It has been reported that nHAC features good biological activity and can promote bone repair and regeneration [8,9,10].

Concentrated growth factor (CGF) is a third-generation platelet concentrate extracted from blood by Sacco in 2006 [11,12]. It is composed of a convenient material, features a wide range of sources, low cost, absorbability, lack of immunogenicity, bone inducibility and other advantages and has attracted increasing attention [13,14]. To promote new bone formation and better meet the needs of oral implants, bone graft materials are often combined with platelet concentrates to enhance bone regeneration [15]. There are reports that the combined use of CGF and bone graft materials can promote osteogenesis and accelerate new bone formation. Wang implanted a mixture of CGF and Bio-oss into the maxillary sinus floor of dogs [16]. Six months after the operation, it was shown that the osteogenesis effect was more significant than that of Bio-oss bone meal alone.

To improve the osteogenic ability of nHAC materials, our research group previously prepared nHAC/CGF materials and cocultured human bone marrow mesenchymal stem cells (hBMSCs) with nHAC and nHAC/CGF materials to assess the adhesion, proliferation and osteogenic differentiation of hBMSCs. Compared with the nHAC material, the nHAC/CGF material features a better ability to promote the adhesion, proliferation and osteogenic differentiation of hBMSCs. However, the timing of bone maturity for successful implantation is still unclear. Further research is needed.

To further explore the ability of nHAC/CGF materials to promote new bone formation and shorten the time of osteogenesis, this study conducted preclinical animal experiments by preparing bone defect rabbit models and implanting nHAC/CGF material and nHAC material into the rabbit model. New bone formation in the bone defect area was observed and analyzed through imaging, histological evaluation and biomechanical testing at 8, 16 and 24 weeks to further clarify whether nHAC/CGF material can accelerate new bone formation and be used as an effective treatment to solve the problem of insufficient bone mass in oral implantation.

## 2. Materials and Methods

### 2.1. Experimental Materials

The nHAC bone graft used in this study is a commercially available artificial bone repair material “BonGold” produced by Beijing Allgens Medical Technology Co., Ltd., Beijing, China. It is composed of layered self-assembled nHA/COL, which features the same composition and microstructure as natural bone and is prepared by an in vitro biomimetic mineralization process.

### 2.2. Experimental Animals

Eighty-one healthy adult New Zealand large-eared rabbits (provided by Qingdao Kangda Biotechnology Co., Ltd., Qingdao, China), with a weight of 3.0 ± 0.5 kg, male and female, were used. The animal experiments were carried out according to the protocol approved by the Welfare Ethics Committee of China Medical University (license number: CMU2019205). All the animals were randomly divided into 8 weeks, 16 weeks and 24 weeks groups, each with 27 animals, all independently fed (feeding was carried out by a breeder from the Laboratory Animal Department of China Medical University, Shenyang, China; healthy animals used as a control were observed for one week. During the surgical procedures, the animals were administered lidocain solution (20 mg/mL), and epinephrine solution (12.5 mg/mL) was used for local anesthesia.

### 2.3. Preparation of CGF

**Figure 1:**
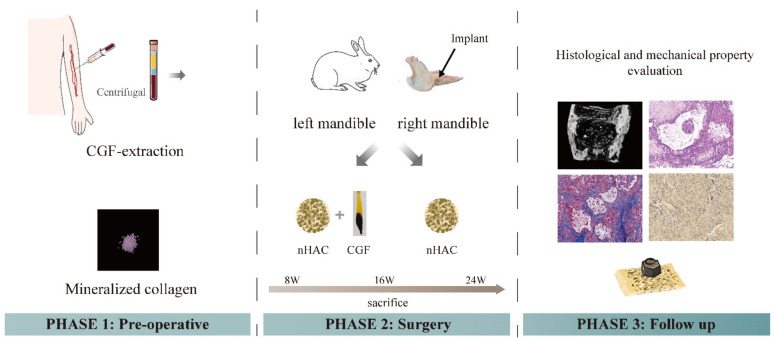
Schematic diagram of the experimental process. The first step was the preparation process of CGF and its mixing with nHAC bone meal. The second step was the implantation process of animal experimental materials. Experimental group, nHAC/CGF; control group, nHAC. In the third step, the detection methods for the materials postoperatively included radiological examination, histological examination and biomechanical examination.

Nine milliliters of experimental rabbit venous blood were extracted into a sterile centrifuge tube without anticoagulant, immediately placed into a centrifuge (Medifuge, Silfradent, Sofia, Italy), and centrifuged according to the CGF program. After centrifugation for 12 min, the venous blood in the test tube was separated into three layers from top to bottom: the upper layer was serum, the middle layer was the fibrin layer (containing a high concentration of CGF), and the bottom layer was red blood cells and platelets. The serum on the top was poured out, while the middle fibrin layer and the adjacent red blood cell layer (the CGF gel) were left intact (Figure 1, phase 1).

### 2.4. Surface Morphology of the Material

After cutting, the CGF gel was mixed with nHAC bone meal at a 1:1 volume ratio, then it was manually mixed and stirred well, pre-frozen in a −80 °C freezer, and then placed in a freeze dryer for 12 h. The surface morphology of nHAC/CGF and nHAC was observed with a scanning electron microscope (S4800, Hitachi, Tokyo, Japan). Before SEM, the surface of the material was plated with a thin layer of gold to provide conductivity.

### 2.5. Establishment of Animal Models and Material Implantation

After the experimental animals were weighed, 9 mL of venous blood were drawn and CGF gel was obtained according to the preparation method in 1.3. It was cut into pieces and mixed with nHAC at a volume ratio of 1:1 for use. Next, the animals were anaesthetized with 5 mL/kg 20% urethane through the ear veins, the rabbits’ bilateral mandibles were depilated and skinned and towels were sterilized. A 2~3 cm parallel incision was made at the lower edge of the mandibular body on both sides. The skin, subcutaneous tissue and fascia were cut layer by layer and the muscles were bluntly separated to expose the bone surface. A hollow bone drill was used to prepare a round bone defect area with a diameter of 8 mm and a depth of 4 mm on the left and right mandibles. In each experimental rabbit bone defect area, the chopped CGF was mixed with nHAC bone meal and implanted on the left side; nHAC was implanted on the right side (Figure 1, phase 2) (*n* = 27 at each time point). Finally, the wound was sutured carefully to ensure postoperative animal welfare. The rabbits were killed by injecting air into the ear vein at 8, 16 and 24 weeks after the operation, and the area of interest was harvested for micro-CT scan, histological analysis and mechanical testing (Figure 1, phase 3).

### 2.6. General Observations

The activity, eating, urination and wound healing of the experimental rabbits in each group were observed after the operation. After the experimental rabbits were sacrificed, changes in the color and volume of the material in the implanted area and bone growth in the defect area were examined; the presence or absence of obvious inflammation were also determined.

### 2.7. Imaging Examination

At 8, 16 and 24 weeks after the operation, the experimental rabbits were sacrificed by ear vein air embolization; the mandibles were removed and the two groups of bone pieces including the new bone, were separated with a wire saw, with a diameter of 8 mm and a depth of 4 mm, and a surrounding normal bone area of 3~5 mm. The specimens were trimmed, washed with normal saline and fixed with 10% neutral formalin for 48 h. Next, X-ray irradiation was performed, the gray value of the bone defect area (diameter of 8 mm) was measured and the grey value was calculated using ImageJ. Next, a micro-CT (Skyscan1276, Bruker, Karlsruhe, Germany) scan was performed, and a cylinder with a diameter of 8 mm and a height of 4 mm was selected as the region of interest (ROI). The scanned slices were reconstructed at high concentrations to show new bone formation and material degradation due to different bone and material thresholds and were analyzed by CT-Analyser software (Bruker, Kontich, Belgium) to assess bone volume (BV), bone volume fraction (BV/TV), trabecular thickness (Tb. Th), trabecular number (Tb. N), bone trabecular separation (Tb. Sp), material volume (MV) and residual volume percentage of material (RMVF).

### 2.8. Histological Observations

At 8, 16 and 24 weeks after surgery and after the X-ray and micro-CT scans, the samples were placed in 10% neutral formalin solution, fixed for 48 h and labelled in groups. The samples underwent step-by-step dehydration, decalcification, embedding and cutting into continuous slices with a thickness of 5 μm. HE staining and Masson staining were used for routine histological evaluation. As for immunohistochemical staining, the specimens were decalcified and then embedded in paraffin. Subsequently, the specimens were cut along the sagittal plane and then deparaffinized. The resulting sections were then incubated with primary antibodies (Anti-BMP-2 antibody, ab6285, Abcam, Cambridge, UK; osteocalcin antibody, NBP2-89037, NOVUS, Littleton, CO, USA) after antigens were recovered using 0.05% parenzyme for 20 min and blocked by BSA. Next, the secondary antibody (Goat Anti-Mouse (ab205719, Abcam, UK) and 3, 3′-diaminobenzidine (DAB, ab64261, Abcam, UK)) was used for secondary staining. Microscopic images of the representative regions were obtained using Axioskop microscopy (Olympus IX71, Tokyo, Japan). The images were acquired using a ToupCam TP610000A microscope acquisition system. For the immunohistochemical analysis, BMP-2 and OCN positive expression area were shown in brown. The average optical density (AOD) of the positive region of three randomly selected high-power fields was measured using an Image-Pro Plus 6.0 software (Image-Pro Plus, Media Cybernetics, Rockville, MD, USA).

### 2.9. Biomechanical Testing

The experimental rabbits were separately sacrificed at 8, 16 and 24 weeks after the operation. A hollow bone drill was used for each sample to completely remove a cylinder of 8 mm (diameter) × 4 mm (height) for mechanical testing. The compression area was π times the square of the radius. At month 0, the test specimens had not undergone surgery. The new bone block specimen was placed vertically on the platform mold of the universal mechanics testing machine, the top of the mechanical machine was pressed to align the specimen and a compression test was performed at a deformation rate of 1 mm/min until it failed. Once it failed, the failure load and displacement of the compression fracture of the bone block were obtained, and the compressive strength and elastic modulus of the new bone block were calculated using the following formulas:

Compressive strength (MPa) = failure load/compression area(1)

Elastic modulus (KPa) = stress/deformation = compressive strength/(displacement/height)(2)

### 2.10. Statistical Analysis

All the experiments were independently repeated more than three times. The data of each group are expressed as the mean ± SD; SPSS 22.0 software was used for data processing. Variance and a chi-square test were used to analyze the data. When *p* < 0.01 for the test parameters and *p* < 0.05 for the nonparametric tests were observed, the differences were considered to be statistically significant.

## 3. Results

### 3.1. The Surface Morphology of the Material

**Figure 2:**
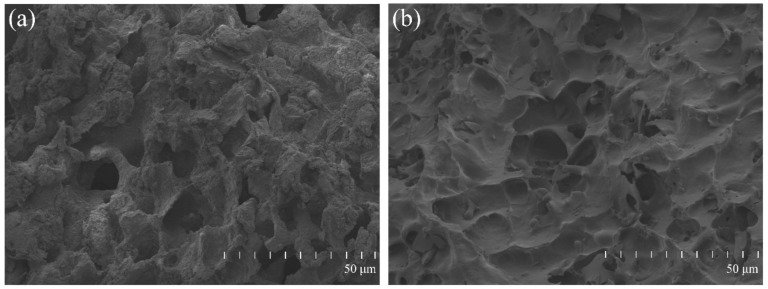
Surface morphology of nHAC/CGF (**a**) and nHAC (**b**) by scanning electron microscopy. The surface of nHAC/CGF was looser, but the pore structure was not changed.

The SEM result in Figure 2 shows that the nHAC material featured different sizes of pores, connected pores and a smooth surface. The nHAC/CGF material maintained the porous structure of the nHAC material and the surface was loose.

### 3.2. General Observation

All the rabbits were anaesthetized and operated on without complications. They recovered well within 12 h and resumed eating. There were no significant changes in appearance or activity, stool characteristics, glandular secretions, body weight, or body temperature before and after surgery. During the six-month follow-up, all the rabbits maintained clinical health and weight. After the general observation of the specimens, undegraded bone meal particles were still visible in the defect area at 8 weeks, with clear boundaries. At 16 weeks, the new bone in each group combined with the surrounding bone. The boundary of the defect area in the nHAC/CGF group was difficult to distinguish and the combination was firm. The boundary in the nHAC group was blurred. At 24 weeks, the two groups displayed no boundary with the surrounding bone. There was no difference between new bone and normal bone in the nHAC/CGF group. At the end of the study, the average body weight ± standard deviation was 4.5 ± 0.3 kg.

### 3.3. Imaging Testing

**Figure 3:**
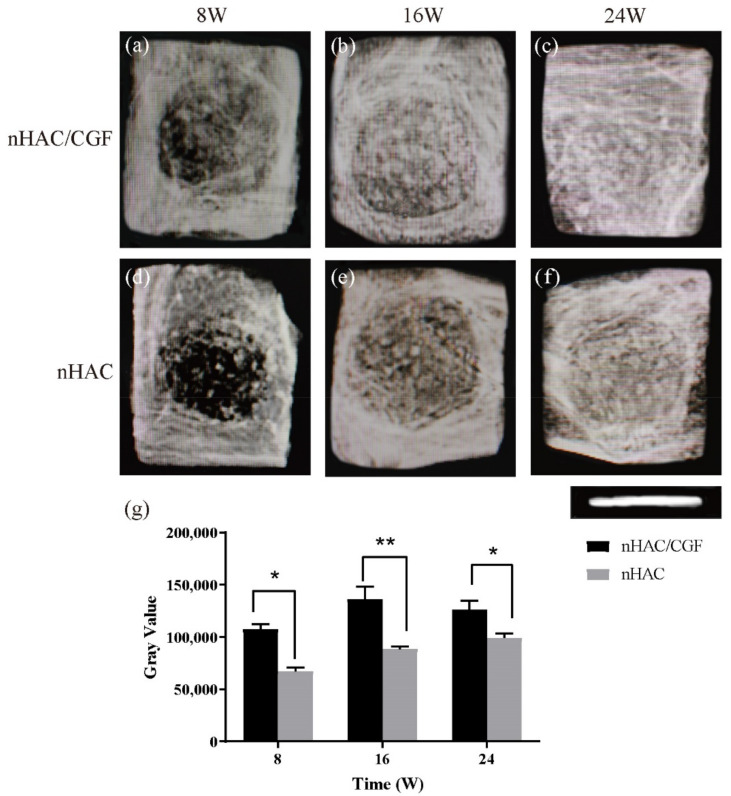
X-ray examination after 8, 16 and 24 weeks of implantation in vivo. (**a**–**f**) X-ray examination of samples of the nHAC/CGF group (**a**–**c**) and nHAC group (**d**–**f**), indicating new bone formation at the defects. (**g**) Integrated optical of the nHAC/CGF group and nHAC group. Ruler length is 8 mm. *n* = 3 in each group; * *p* < 0.05, ** *p* < 0.01.

At 8, 16 and 24 weeks after surgery, the X-ray films (Figure 3) showed a downward trend along with material degradation and an upward trend in new bone formation, and the bone mineral density of the two groups in the defect areas gradually increased. At the same time, the amount of new bone formation in the nHAC/CGF group was significantly greater than in the nHAC group (*p* < 0.05). At 8 weeks, the bone defect area in the nHAC/CGF group exhibited obvious bridging, and a small amount of new bone was formed, while the bone defect area in the nHAC group was cloudy, with less new bone formation. At 16 weeks, as most of the material in the nHAC/CGF group was degraded, the defect area was filled with new bone. At 24 weeks, the material in the bone defect area in the nHAC/CGF group was almost completely degraded, and the bone density of the new bone was not significantly different from that of normal bone tissue. The bone density of the nHAC group was still lower than that of the normal bone tissue at 24 weeks and was close to that of the nHAC/CGF group at 16 weeks.

**Figure 4:**
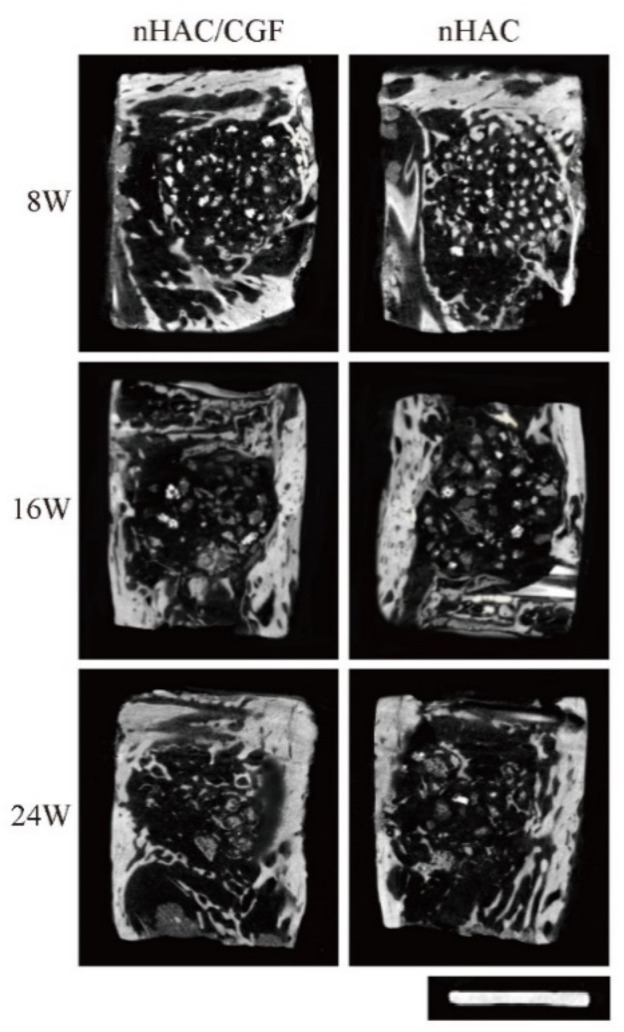
Micro-CT of the two-dimensional reconstruction image. Representative two-dimensional reconstruction cross-sections of micro-CT scans at 8, 16 and 24 weeks after surgery. Ruler length is 8 mm.

**Figure 5:**
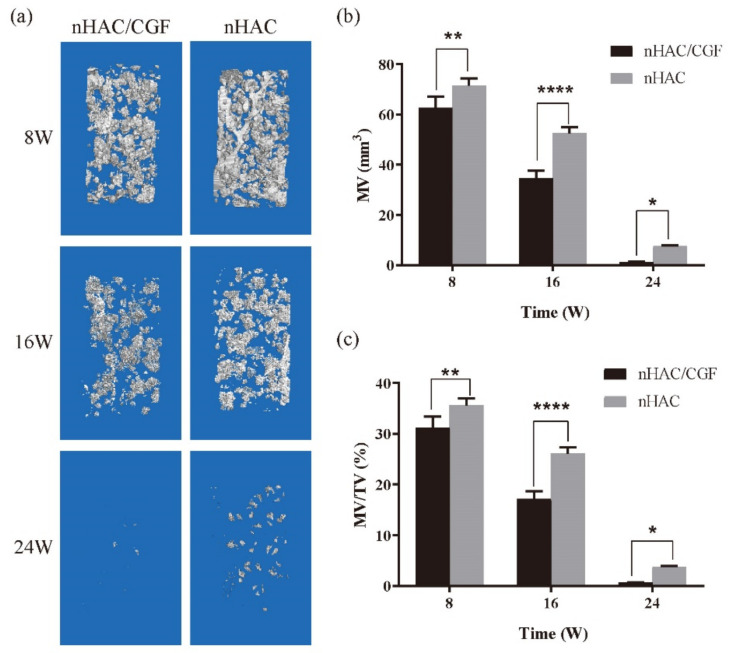
Degradation of materials in vivo. (**a**) The residue of material in the ROI at 8, 16 and 24 weeks from the nHAC/CGF and nHAC groups. (**b**,**c**) The changes in material volume during degradation after surgery in each group. *n* = 3 in each group; * *p* < 0.05, ** *p* < 0.01, **** *p* < 0.0001.

**Figure 6:**
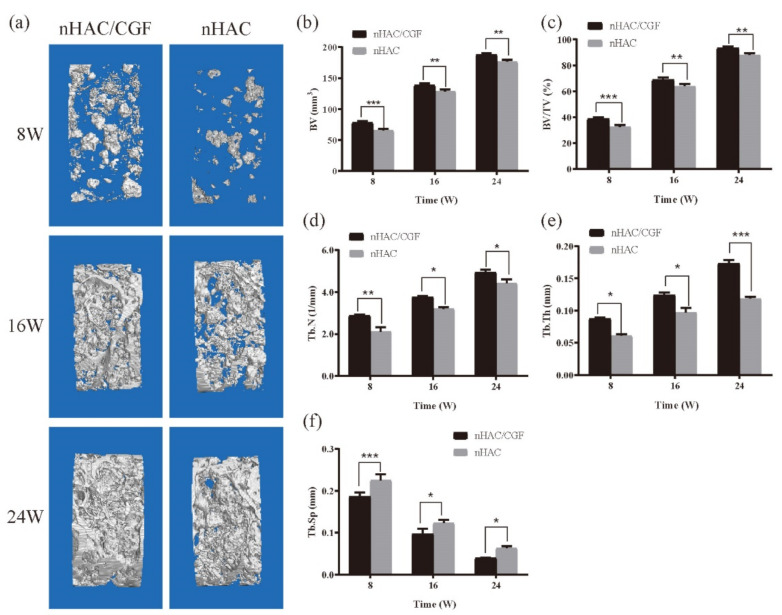
New bone formation in vivo. (**a**) Representative 3D micro-CT images within the ROI at 8, 16, and 24 weeks after surgery. (**b**–**f**) Quantitative analysis of micro-CT of the new bone in the ROI.: (**b**) BV; (**c**) BV/TV; (**d**) Tb.N; (**e**) Tb.Th; (**f**) Tb.Sp. *n* = 3 in each group; * *p* < 0.05, ** *p* < 0.01, *** *p* < 0.001.

The micro-CT results visually showed the difference between the bone regeneration process of the two groups at different time points (Figure 4). The representative two-dimensional cross-sectional view in Figure 4 confirms that the nHAC/CGF group demonstrated better new bone formation and material degradation in the bone defect area at 8, 16 and 24 weeks after surgery. The results of the micro-CT three-dimensional reconstruction analysis (Figure 5 and Figure 6) showed that 8 weeks after surgery, compared with the nHAC group (BV 65.47 ± 2.83 mm^3^, MV 74.48 ± 2.82 mm^3^, Tb.Th 0.06 ± 0.00 mm, Tb.N 2.12 ± 0.20 1/mm, Tb.Sp 0.23 ± 0.01 mm), the nHAC/CGF group’s BV (78.00 ± 2.68 mm^3^), Tb.Th (0.09 ± 0.00 mm), and Tb.N (2.87 ± 0.06 1/mm) were higher and the MV (62.58 ± 4.67 mm^3^) and Tb.Sp (0.19 ± 0.01 mm) were lower. At 16 weeks postoperatively, the average new bone volume in the nHAC/CGF group was 138.75 ± 3.25 mm^3^, which reached 69% of the total volume. At 24 weeks, the nHAC/CGF group almost reached bone maturity, with a BV/TV of 93.34 ± 1.30%; the material was almost completely degraded (MVF 0.61 ± 0.16 mm^3^).

### 3.4. Histological Evaluation

**Figure 7:**
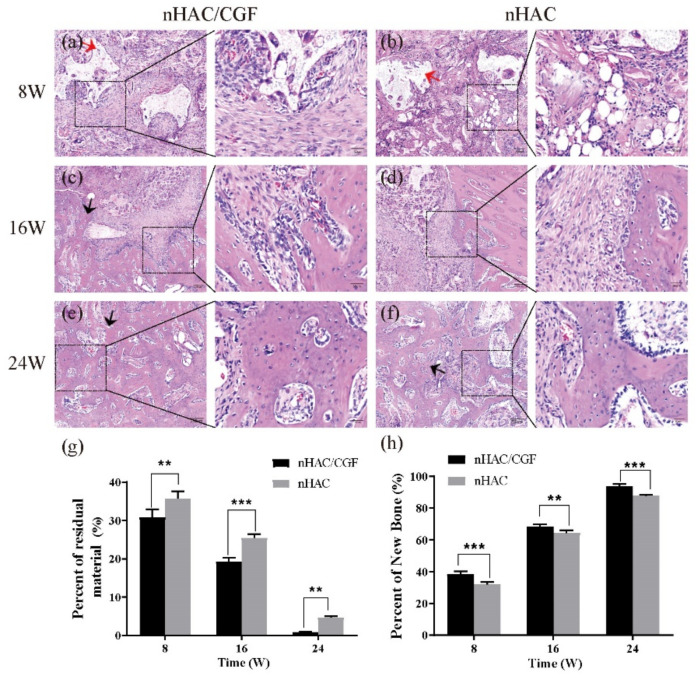
Qualitative and quantitative analysis of HE staining. (**a**–**f**) New bone formation and material degradation in the defect area at 8, 16, and 24 weeks after implantation of the nHAC/CGF mixture and the nHAC alone. (**g**,**h**) Quantitative analysis results of the percentage of new bone formed in the defect area and the percentage of material remaining. *n* = 3 in each group; ** *p* < 0.01, *** *p* < 0.001. Red arrow: material; black arrow: new bone.

**Figure 8:**
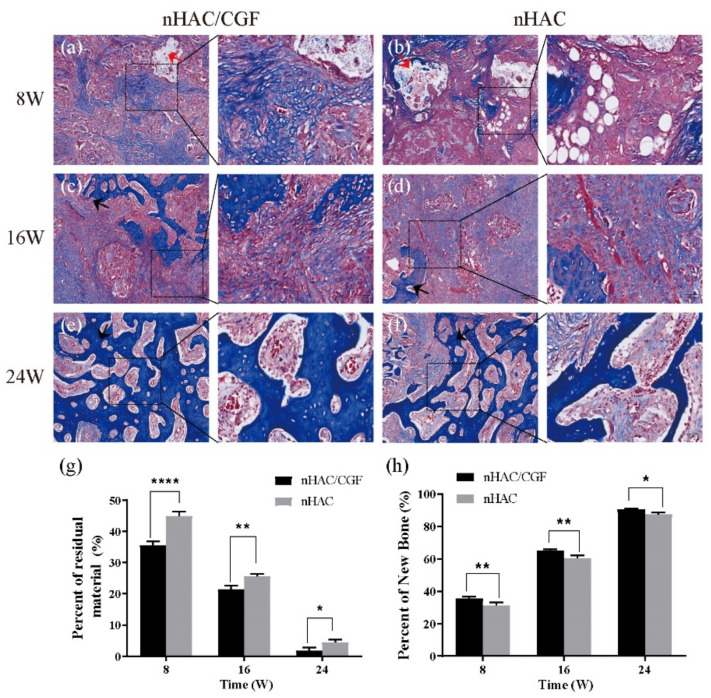
Qualitative analysis of Masson staining. (**a**–**f**) New bone formation and material degradation in the defect area at 8, 16 and 24 weeks after implantation of the nHAC/CGF mixture and the nHAC alone. *n* = 3 in each group. (**g**,**h**) Quantitative analysis results of the percentage of new bone formed in the defect area and the percentage of material remaining. *n* = 3 in each group; * *p* < 0.05, ** *p* < 0.01, **** *p* < 0.0001. Red arrow: material; black arrow: new bone.

The histological evaluation results of the HE staining (Figure 7a–f) and Masson staining (Figure 8a–f) both showed that new bones were formed to different degrees and that the materials were degraded to different degrees 8, 16 and 24 weeks after surgery. At 8 weeks, the nHAC group saw more material remaining, aggregated fat cells, scattered new bone formation and a small amount of fibrous tissue, while the nHAC/CGF group saw a small amount of new bone formation, more fibrous tissue, scattered fat cells and blood vessel formation. At 16 weeks, the boundary between the defect area and normal bone tissue in the nHAC/CGF group was blurred, showing mature bone tissue, while the boundary between the defect area and normal bone tissue in the nHAC group was still clear. At 24 weeks, the new bone in the nHAC/CGF group was not significantly different from the surrounding normal bone and there was no difference in structure. The boundary between the new bone and the surrounding normal bone in the nHAC group was unclear, but the structure was scattered and uneven, immature bone tissue was still visible and almost no material remained. Quantitative histological statistics showed that compared with the nHAC group, the nHAC/CGF group featured a higher percentage of new bone formation and less material remaining. The quantitative results of HE staining in the nHAC/CGF group and nHAC group were 38.42 ± 1.70% and 32.08 ± 1.48% at 8 weeks, 68.57 ± 1.11% and 64.29 ± 1.72% at 16 weeks and 93.61 ± 1.53% and 87.77 ± 0.75% at 24 weeks, respectively. The quantitative results of the Masson staining in the nHAC/CGF group and nHAC group were 35.51 ± 1.16% and 31.29 ± 1.94% at 8 weeks, 65.17 ± 0.80% and 60.57 ± 1.46% at 16 weeks and 90.42 ± 0.67% and 87.47 ± 1.12% at 24 weeks, respectively.

**Figure 9:**
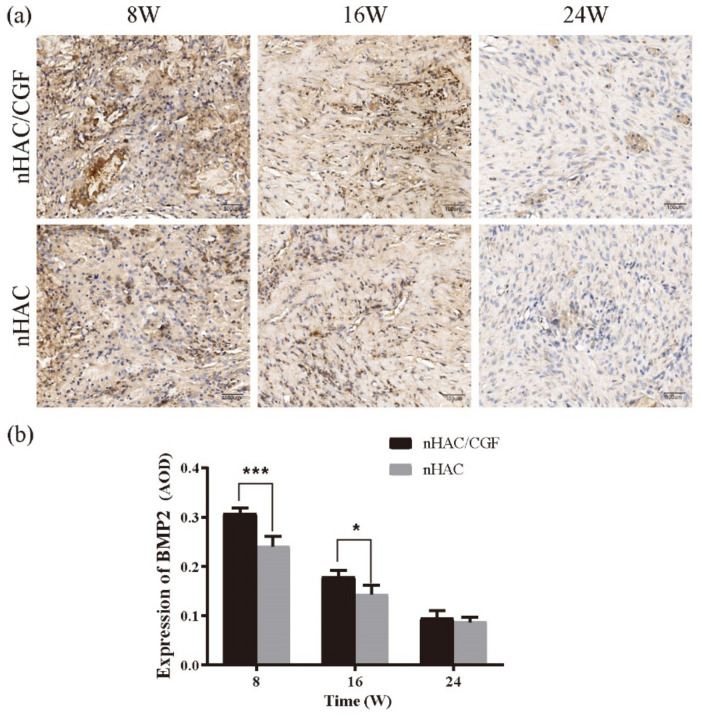
Immunohistochemical analysis of BMP2 after implantation. (**a**) BMP2 expression levels were detected by immunohistochemical evaluation, and positively stained brown cells were seen. (**b**) Quantitative analysis of BMP2 levels expressed in new bone formation at 8, 16 and 24 weeks after implantation. *n* = 3 in each group; * *p* < 0.05, *** *p* < 0.001.

**Figure 10:**
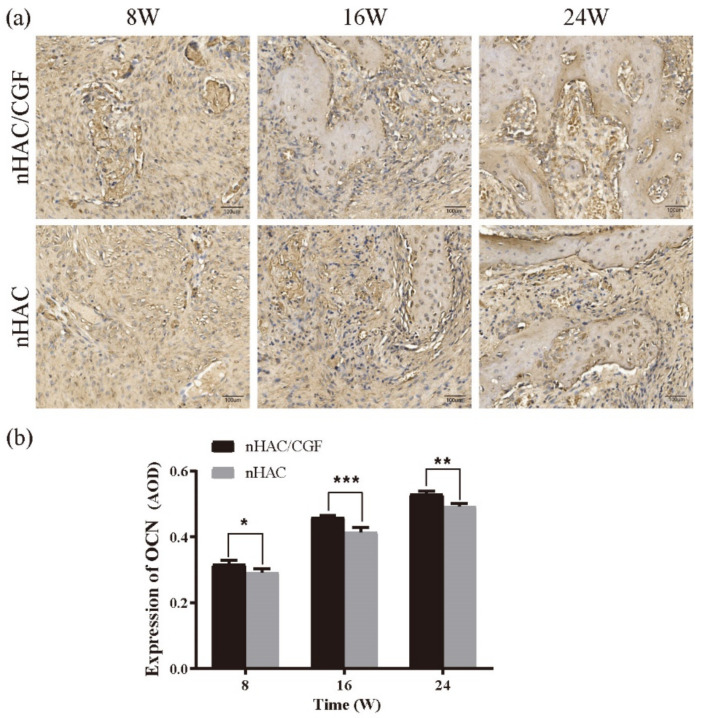
Immunohistochemical analysis of OCN after implantation. (**a**) OCN expression levels were detected by immunohistochemical evaluation and positively stained brown cells were seen. (**b**) Quantitative analysis of OCN levels in new bone formation at 8, 16 and 24 weeks after implantation. *n* = 3 in each group; * *p* < 0.05, ** *p* < 0.01, *** *p* < 0.001.

The expression of BMP2 and OCN in the nHAC/CGF group and the nHAC group is shown in Figure 9 and Figure 10. At 8 weeks after surgery, the positive expression of BMP2 in the nHAC/CGF group was significantly higher than that in the nHAC group, but the expression decreased with time. At 16 weeks, the positive expression values between the two groups were relatively close, and the difference was statistically significant (*p* < 0.05). There was almost no expression at 24 weeks, and the difference between the two groups was not statistically significant (*p* > 0.05). Over time, the expression of OCN increased significantly. At 8 weeks postoperatively, the two groups featured a small amount of positive OCN expression, while the difference between the two groups was statistically significant (*p* < 0.05). At 16 weeks postoperatively, the positive expression of OCN in the nHAC/CGF group was significantly higher than that in the nHAC group (*p* < 0.0001) and a small amount of new bone formation was seen. After 24 weeks, the expression of OCN in the two groups was still increasing. The new bone in the nHAC group was loosely formed, while the new bone in the nHAC/CGF group was basically mature and filled the defect area.

### 3.5. Biomechanical Testing

**Figure 11:**
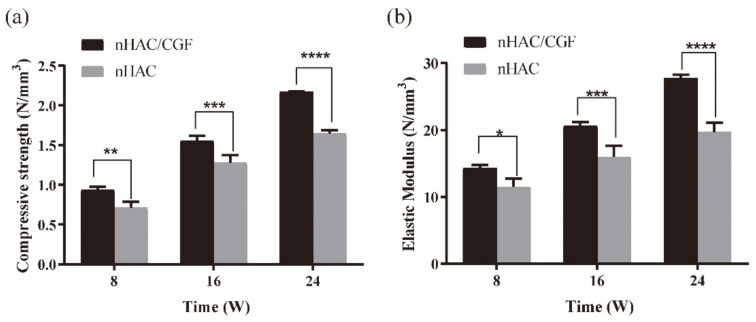
Biomechanical testing in vivo. Compressive strength (**a**) and elastic modulus (**b**) at 8, 16 and 24 weeks after surgery. *n* = 3 in each group; * *p* < 0.05, ** *p* < 0.01, *** *p* < 0.001, **** *p* < 0.0001.

Figure 11 shows the results of the biomechanics testing. After the compression strength test was performed at 8, 16 and 24 weeks after the operation, the displacement and maximum load values were obtained, and the compressive strength and elastic modulus were calculated by formulas. As time increased, the intensity of new bone formation gradually increased, and the difference between the two groups was statistically significant. The strength of the nHAC group (compressive strength of 0.71 ± 0.08 MPa and elastic modulus of 11.48 ± 1.25 MPa) was the lowest at 8 weeks postoperatively, and the strength of the nHAC/CGF group (compressive strength of 0.94 ± 0.04 MPa and elastic modulus of 14.32 ± 0.48 MPa) strength was slightly higher. The compressive strength (1.28 ± 0.10 MPa) and elastic modulus (15.96 ± 1.74 MPa) of the nHAC group at 16 weeks were close to those of the nHAC/CGF group at 8 weeks but significantly lower than those of the nHAC/CGF group. The strength of the two groups increased significantly at 24 weeks after surgery. The compressive strength (2.17 ± 0.16 MPa) and elastic modulus (27.72 ± 0.59 MPa) of the new bone formation in the nHAC/CGF group were the same or close to those of normal bone (compressive strength of 2.17 ± 0.05 MPa, modulus of elasticity of 28.26 ± 0.46 MPa).

## 4. Discussion

nHAC is a new type of bone tissue engineering scaffold material. The in vitro preparation process of nHAC mimics natural bone formation. Type-I collagen fibrils act as template providing sites for the nucleation and growth of the nanocrystals HA [17]. Researchers have found that the hierarchical structure of nHAC is similar to the nanostructure of natural bone [18]. Therefore, nHAC offers promising biocompatibility and osteoconductivity to promote new bone regeneration [19,20].

Many studies have shown that the combined application of CGF and bone substitute materials can promote osteogenesis [21,22]. To explore whether CGF can promote the osteogenesis of nHAC effectively, we explored the behavior of hBMSCs cultured on nHAC/CGF in vitro. The results showed that, compared with nHAC materials, nHAC/CGF materials can promote the adhesion, proliferation and osteogenic differentiation of hBMSCs effectively. To verify whether the nHAC/CGF material can effectively promote bone formation in vivo and shorten the bone formation time, in this study, we implanted the nHAC/CGF material and the nHAC material into the rabbit mandibular defect model and performed imaging, histological and biomechanical testing. The results showed that the nHAC/CGF material features a better and faster osteogenic ability and better biomechanical properties than the nHAC material.

Micro-CT can accurately and objectively evaluate bone structure and provide comprehensive bone parameters [23,24]. Therefore, in our experiment, we used it in combination with X-rays for imaging. Both the X-ray and Micro-CT results show that the nHAC/CGF material features a better new bone formation ability in the bone defect area. Micro-CT two-dimensional and three-dimensional reconstruction of the new bone in the defect area and analysis of BV, BV/TV, RMV, RMVF, Tb.Th, Tb.N, Tb.Sp and other parameters were measured at 8, 16 and 24 weeks after surgery. With the increase in time, compared with the nHAC group, the nHAC/CGF group demonstrated material degradation, a faster formation of new bone, and a greater amount of new bone. By 24 weeks, the nHAC/CGF material was completely degraded and the new bone was filled with the defect area.

The results of this study are similar to those of other scholars [25,26]. Topkara implanted CGF and autologous bone under the skin of rabbits, indicating that CGF and autologous bone can enhance new bone formation when used in combination [27]. After establishing an animal model, Durmuşlar found that the combination of CGF and autologous bone or Bio-oss can promote bone regeneration and shorten the time for new bone formation [28].

The detection methods used in this study were comprehensive; the bone parameters were diverse, which is widely accepted by other studies [29]. The results confirmed that the new bone in the nHAC/CGF group was mature at 24 weeks, which may be of guiding significance for the selection of the appropriate time for clinical implantation.

The ideal scaffold material must feature a controllable biodegradation rate corresponding to the bone remodeling rate [30]. Our study showed the same results. The histological evaluation results showed that in the early implantation area, more new bone formation and less material were observed in the nHAC/CGF group than in the nHAC group. At 8 weeks, the nHAC/CGF group featured a small amount of fat cells and more fibrous tissue and blood vessel formation. Trabecular bone structure and mature bone tissue could be seen at 16 weeks, as well as mature bone at 24 weeks in the late stage, while immature bone was still seen in the nHAC group at 24 weeks.

BMP2 is the early signaling molecule of bone morphogenesis and can induce bone marrow mesenchymal stem cells to differentiate into osteoblasts [31]. The results of the immunohistochemical staining showed that in the presence or absence of CGF, especially in the early stage, the expression of BMP2 was different, with the expression highest at 8 weeks and decreasing with time. Similar results were seen at 16 weeks, while almost no expression was observed at 24 weeks. From the initial bone formation to the end of the 24-week observation period, the results reached the bone level as expected, which is consistent with the bone formation trend of BMP2 in vivo discussed by Lai [32].

OCN is produced by osteoblasts [33]. The level of OCN can directly reflect the formation and reconstruction of bone tissue [34]. It is an important sign of mature bone [35]. It is generally expressed from the early stage of calcification. The nodules reach a peak after they mature [36]. From the experimental results, the positive results of the OCN immunohistochemical staining showed that the new bone formation in the defect area was small at 8 weeks, increased at 16 weeks and highest at 24 weeks. The difference between the two groups was statistically significant (*p* < 0.05), which is consistent with the conclusion of the high expression of OCN in late osteogenesis [37]. On the basis of the imaging test results, our histological evaluation results further confirmed that CGF can effectively promote new bone formation of nHAC, and the longer experimental period clarifies the exact time for bone maturity, in that at 24 weeks, the nHAC/CGF group featured mature bone, but the nHAC group still had immature bone.

During the degradation and absorption of bone tissue engineering scaffold materials, the strength of the scaffold should remain unchanged until the implanted area is completely reshaped by the host tissue and its structural role is fully exerted [38,39]. In our study, through imaging and histological evaluations, it was confirmed that nHAC/CGF materials feature good new bone formation and material degradation characteristics, but the exact time of bone maturity and whether the new bone can support implant repair were still unknown. Therefore, we performed biomechanical testing on the new bone at 8, 16 and 24 weeks after surgery to obtain the compressive strength and elastic modulus, which were compared with normal bone (compression strength of 2.18 ± 0.01 MPa, elastic modulus of 28.26 ± 0.46 MPa) to determine the appropriate implantation time point. Qiu conducted mechanical tests on new bone after nHAC bone meal was implanted into the bone defect; the compressive strength and elastic modulus were shown to gradually increase with time [40]. The nHAC group in our experiment showed the same results. The nHAC/CGF group was higher than the nHAC group at all time points. By 24 weeks, its compressive strength (2.17 ± 0.16 MPa) and elastic modulus (27.72 ± 0.59 MPa) were the same as those of normal bone. The results showed that the addition of CGF promoted the osteogenesis of nHAC and shortened the osteogenesis time. At the same time, the biomechanical performance of the nHAC/CGF group was better than that of the nHAC group. At 24 weeks, the strength of the new bone reached the normal bone level, which can provide a theoretical basis for nHAC to increase bone mass in implants.

Our research results show that the nHAC/CGF material can accelerate the formation of new bone, shorten the time of bone formation and provide a theoretical basis for insufficient bone mass in the field of oral implants. However, the mechanism of nHAC/CGF in promoting bone formation is still unclear and further research is needed. Our research team will undertake in-depth research and discussions in future experiments.

## 5. Conclusions

Through imaging, histological and biomechanical testing methods, it was demonstrated that compared with nHAC materials, nHAC/CGF materials show good new bone formation ability in the implanted rabbit mandibular defect model, reaching bone maturation at 24 weeks. It was confirmed that the use of CGF can promote new bone formation in nHAC and that new bone features the same bone strength as normal bone tissue at 24 weeks. This study provides a theoretical basis for the application of this new type of bone tissue engineering scaffold material in terms of insufficient bone mass in oral implants to increase bone mass and can be used to guide clinical practice.

## Data Availability

The data presented in this study are available on request from the corresponding author.
